# 

**Published:** 1900-03-10

**Authors:** 


### NOTICE TO CORRESPONDENTS.

All MS., Letters, Books for Review, and other matters intended for the Editor should be addressed The Editor, "The Hospital," The Hospital Building, 28 & 29, Southampton Street, Strand, W.C.

The Editor cannot undertake to return rejected MS., even when accompanied by stamped directed envelope.

**Notice to Advertisers and Subscribers.**

All Advertisements, Orders for Copies of The Hospital, and Business Communications should be addressed to **THE MANAGER (Not to the Editor),** The Hospital, The Hospital Building, 28 & 29, Southampton Street, Strand, London, W.C.

The Cost of Subscription, post free, is as follows:—Per week, 2½d.; per quarter, 2s. 8d.; per half-year, 5s. 3d.; per annum, 10s. 6d. Foreign:—Per annum, 15s. 2d., payable, in all cases, in advance.

**NOTICE.—Vol. XXVI. of "The Hospital" (April, 1899, to September, 1899), in handsome cloth binding, is now on sale at the Office of this Journal, price 7s. 6d. Cases for binding the Numbers contained in this Volume 1s. 6d. Index to Vol. XXVI., 2½d., post free.**

**The Monthly Part for March is now ready. Price 6d., by post 8d.**

### THE STUDENT OF MEDICINE.

### APPOINTMENTS.

C. M. Allan, M.A., M.D., M.S.Edin., appointed Public Vaccinator for the Luyton District of the Stoke-upon-Trent Union. G. H. S. Blackburne, M.B., B.S., appointed House Physician to the Perth Hospital, West Australia. J. H. Brooks, M.D., M.B., C.M.Aberd., appointed Medical Superintendent of the Mile End Infirmary, and Medical Officer of the Mile End Workhouse and Schools. H. M. Cockcroft, L.R.C.P., L.R.C.S.Edin., L.F.P.S.Glasg., appointed Medical Officer and Public Vaccinator for the Masham District of the Leyburn Union. W. B. Docker, M.B., appointed Health Officer for the Borough of Portland, Victoria. T. Dunlop, M.B., C.M.Edin., D.P.H.Camb., appointed Medical Officer of Health to the Aldershot Urban District Council. D. H. E. Lines, M.B., Ch.B.Melb., appointed Assistant House Surgeon to the Hobart General Hospital. W. W. Linington, F.R.C.S.Eng., appointed Honorary Assistant Medical Officer to the Victoria Hospital, Folkestone. W. B. Mercer, M.B., D.P.H.Camb., M.R.C.S., L.R.C.P., appointed Medical Officer of Health to the Rushworth Urban District Council. P. Murison, M.B., C.M.Edin., appointed Medical Officer of Health for Sutherlandshire. S. Prior, M.B., C.M.Glasg., appointed Medical Officer of the Kirkheaton District of the Huddersfield Union. W. G. Richardson, M.B., B.S.Dunelm., F.R.C.S.Eng., appointed Assistant Surgeon to the Royal Infirmary, Newcastle-on-Tyne. R. H. Ritchie, M.B., appointed Health Officer for the Borough of Horsham, Victoria. Ella C. Scarlett, M.D.Brux., L.M., L.S.A., appointed Assistant Surgeon to the Household of H.M. the Emperor of Korea. E. J. Steegmann, M.B., D.P.H., appointed Assistant Lecturer on Hygiene and Public Health at St. Mary's Hospital Medical School. J. Thompson, M.B., B.S., appointed Resident Medical Officer to the Perth Public Hospital, Western Australia.

Demy 8vo., with Portrait, cloth gilt, 16s.

**GEORGE HARLEY, F.R.S.; or The Life of a London Physician.** By Mrs. ALEC TWEEDIE.

"The authoress is well known by her pleasant and chatty books of travel. . . She has succeeded, by a judicious combination of her father's notes with her own recollections, in producing a readable and interesting memoir."—*The Times.*

LONDON: THE SCIENTIFIC PRESS, LTD., 28 & 29, Southampton Street, Strand, W.C.

### NOW READY,

A REPRINT OF

### A NURSING HANDBOOK

TOGETHER WITH S. MAW, SON, AND THOMPSON'S

### COMPLETE CATALOGUE OF NURSING REQUISITES.

The above work is now republished at a cost of 1s. per copy. Messrs. S. M., S., and T. have been compelled to make this charge on account of the extreme popularity of the work and the great expense incurred in its production.

***Post Free on receipt of 1s.***

### S. MAW, SON, and THOMPSON,

7 to 12, ALDERSGATE STREET, LONDON, E.C.

